# Supplementary material for: Increasing young people’s use of sexual and reproductive health services in government health facilities in rural Kenya
Source: PLoS One. 2026 May 4;21(5):e0347329. doi: 10.1371/journal.pone.0347329 (PMC13138647; doi:10.1371/journal.pone.0347329)
Supplement: S1 File — (DOCX) [file pone.0347329.s004.docx]

Inclusivity in global research

# Ethical considerations, permits and authorship

*This section is applicable to all research types.*

Provide details as to who granted permissions and/or consent for the study to take place in the Methods section of your manuscript. This should include the names of **all** ethics boards, governmental organizations, community leaders or other bodies that provided approval for the study. If individuals provided approval refer to these people by their role or title but do not list their name(s).

The implementing agency, CSA, obtained approval from the County Government of Bungoma - Department of Health to conduct AHAP. Ms. Milsane Kiplai, the county's adolescent sexual and reproductive health, gender-based violence and early childhood development coordinator, signed the approval letter.

If there were any deviations from the study protocol after approval was obtained please provide details of these changes in the Methods section of your manuscript.

N/A (not applicable because no deviations were made)

Did this study involve local collaborators that are residents of the country where the research was conducted or members of the community studied? If you do not have any authors from said communities, please provide an explanation for this below.

Yes, the study involved Kenyan local collaborators who are co-authors on the paper (Albert Obbuyi and Collins Juma). At the time of the study, Mr. Obbuyi was the head of the implementing NGO, CSA, and Mr. Juma was the Project Coordinator. They reviewed drafts and gave extensive edits to the manuscript.

Everyone listed as an author should meet PLOS’ criteria for authorship and all individuals who meet these criteria should be included in the author byline, rather than the acknowledgements. For further information please see the journal’s Authorship Policy.

# Human subjects research (e.g. health research, medical research, cross-cultural psychology)

Did you obtain written informed consent from a representative of the local community or region before the research took place? How did you establish who speaks for the community? Details of written informed

As stated earlier, CSA received written approval from the County Government of Bungoma - Department of Health to conduct the project.

consent obtained from study participants should be reported separately in the Methods section of your manuscript.

How did members of the local community provide input on the aims of the research investigation, its methodology, and its anticipated outcome(s)?

CSA conducted a 2-hour inception meeting with key stakeholders who included: county health officials, health service providers from the selected facilities, school principals and guidance counselors, religious leaders, community leaders and youth representatives. All were presented with the proposed plan for the project and study. During the course of the meeting, they provided useful feedback which was used in the implementation of the project and study. For example, the youth representatives suggested that all AHAP facilities receive board games and youth-oriented SRH brochures and posters, which we added to the project plan. Also, service delivery hours and improvements to be made to the health facilities were discussed, and suggestions provided by local health management teams and the local youth were incorporated into the AHAP intervention design.

When engaging with the local community, how did you ensure that the informed consent documents and other materials could be understood by local stakeholders?

The only informed consent documents we used were for AHAP nurses. Prior to being included in AHAP and trained in comprehensive sexuality education, they were informed of what their duties would be and their compensation if they were to be part of AHAP. They were also informed that they would need to take

pre-test and post-test surveys. Since the nurses spoke English fluently (nursing school is in English), the documents were in English.

Will the findings of the research be made available in an understandable format to stakeholders in the community where the study was conducted (e.g. via a presentation, summary report, copies of publications, etc.)? Please provide details of how this will be achieved.

In June 2019, at the conclusion of the evaluation, we had a 3-hour dissemination workshop in the local area (Bungoma County, Kenya) with about 45 community stakeholders (health officials, education officials, nurses, young people, CBO representatives in Bungoma). We spent an hour presenting the findings, then broke into small groups for another hour to discuss the implications of the findings, before returning to the plenary for sharing out.

# Non-human subjects research using specimens/ animals collected as part of the study, or those housed in archival collections. Examples include archaeology, paleontology, botany and zoology.

Did the permission you obtained from a local authority to perform the study include an agreement on access to outputs and benefit sharing? This may include procedures to enable fair distribution of the benefits and resources arising from the research performed. Please include any details of Prior Informed Consent and Benefit Sharing Agreements obtained. These may be required by field-specific regulations, for example the Convention on Biological Diversity (CBD) and the associated Nagoya Protocol.

N/A

If the material used in your study was imported, please A) provide the year it was imported and B) indicate whether permits were obtained to import/export the materials used, C) provide details of any permits obtained. If this information is not available, please indicate this.

N/A

If you used archival specimens, please state how the material used in your study was acquired by the institute it is held in and provide details of any permits obtained for the original excavations/ sample collection. If this information is not available, please indicate this.

N/A

How was the potential cultural significance of the materials collected in your study to local communities considered in your research design? Were Indigenous peoples and/or local researchers and institutions involved with archaeological excavations / collection of specimens? If so, please provide a description of their involvement.

N/A

If your manuscript includes photographs of human remains please indicate whether authors obtained permission from descendants or affiliated cultural communities to do so.

N/A
