# Supplementary material for: Increasing young people’s use of sexual and reproductive health services in government health facilities in rural Kenya
Source: PLoS One. 2026 May 4;21(5):e0347329. doi: 10.1371/journal.pone.0347329 (PMC13138647; doi:10.1371/journal.pone.0347329)
Supplement: Attachment — Submitted filename: PONE-D-25-35983-AF.pdf [file pone.0347329.s005.pdf]

PLOS ONE

Increasing young people's use of sexual and reproductive health services in government health facilities in rural Kenya

--Manuscript Draft--

Manuscript Number:	PONE-D-25-35983
Article Type:	Research Article
Full Title:	Increasing young people's use of sexual and reproductive health services in government health facilities in rural Kenya
Short Title:	Increasing adolescent use of reproductive health services in Kenya
Corresponding Author:	Paula Tavrow, PhD<br>University of California Los Angeles<br>Los Angeles, CA UNITED STATES OF AMERICA
Keywords:	adolescents; Kenya; sexual and reproductive health; Rural; health facilities; Access; adolescent and youth friendly services
Abstract:	Young people in sub-Saharan Africa, especially in rural areas, face numerous geographic and psychosocial barriers to accessing sexual and reproductive health (SRH) services and information. Services are rarely offered at convenient times and providers can be judgmental. Adolescents worry that they will not be accorded privacy and confidentiality. They are also concerned that community members or extended family members may see them waiting for SRH services, which could lead them o being perceived as promiscuous and incur parental punishment. Because they lack comprehensive sexuality education (CSE) in schools, they have serious misconceptions about contraceptives. To address these barriers and increase youths' uptake of SRH services, the After-Hours Adolescent Project (AHAP) was created as a low-cost intervention for rural government facilities. The main strategies involved extending clinic hours into early evening to be more convenient and confidential for youths, and training newly-graduated nurses to serve as AHAP nurses. AHAP was tested using a randomized cluster design in thirteen government health facilities in Western Kenya. The study examined two intervention types: (1)t he full intervention, which included having AHAP nurses conduct CSE classes in nearby schools and community locations, and (2) a partial intervention, which was entirely clinic-based. After one year, AHAP increased young people's use of SRH services by 87% (full intervention 97%; partial intervention 77%), whereas comparison facilities saw no change. The full intervention facilities also attracted significantly more female and younger SRH clients. Additionally, improved nurse SRH attitudes after training were sustained. In sum, AHAP was acceptable and highly effective in rural settings, but requires ongoing budget outlays for nurse salaries.
Order of Authors:	Paula Tavrow
	Krupa Maria Varghese
	Albert Obbuyi
	Collins Juma
Opposed Reviewers:	
Additional Information:	
Question	Response
**Financial Disclosure**<br><br>Enter a financial disclosure statement that describes the sources of funding for the work included in this submission. Review the submission guidelines for detailed requirements. View published research	Yes

Powered by Editorial Manager® and ProduXion Manager® from Aries Systems Corporation

articles from *PLOS ONE* for specific examples.<br><br>This statement is required for submission and **will appear in the published article** if the submission is accepted. Please make sure it is accurate.<br><br>**Funded studies**<br>Enter a statement with the following details:<br>• Initials of the authors who received each award<br>• Grant numbers awarded to each author<br>• The full name of each funder<br>• URL of each funder website<br>• Did the sponsors or funders play any role in the study design, data collection and analysis, decision to publish, or preparation of the manuscript?<br><br>Did you receive funding for this work?	
Please add funding details.<br>as follow-up to "**Financial Disclosure**<br><br>Enter a financial disclosure statement that describes the sources of funding for the work included in this submission. Review the submission guidelines for detailed requirements. View published research articles from *PLOS ONE* for specific examples.<br><br>This statement is required for submission and **will appear in the published article** if the submission is accepted. Please make sure it is accurate.	Lalor Foundation and Save the Children (Sweden) supported the program implementation, which was in this study. Both funders had no role of any kind in study design, data collection, analysis, etc., and did not specifically fund the evaluation.

**Funded studies**<br>Enter a statement with the following details:<br>• Initials of the authors who received each award<br>• Grant numbers awarded to each author<br>• The full name of each funder<br>• URL of each funder website<br>• Did the sponsors or funders play any role in the study design, data collection and analysis, decision to publish, or preparation of the manuscript?<br><br>Did you receive funding for this work?"	
Please select the country of your main research funder (please select carefully as in some cases this is used in fee calculation).<br>as follow-up to "**Financial Disclosure**<br><br>Enter a financial disclosure statement that describes the sources of funding for the work included in this submission. Review the submission guidelines for detailed requirements. View published research articles from *PLOS ONE* for specific examples.<br><br>This statement is required for submission and **will appear in the published article** if the submission is accepted. Please make sure it is accurate.<br><br>**Funded studies**<br>Enter a statement with the following details:<br>• Initials of the authors who received each award<br>• Grant numbers awarded to each author<br>• The full name of each funder<br>• URL of each funder website<br>• Did the sponsors or funders play any role in the study design, data collection and analysis, decision to publish, or preparation of the manuscript?<br><br>Did you receive funding for this work?"	UNITED STATES - US
**Competing Interests**	The authors have declared that they have no competing interests.

Use the instructions below to enter a competing interest statement for this submission. On behalf of all authors, disclose any competing interests that could be perceived to bias this work—acknowledging all financial support and any other relevant financial or non-financial competing interests.<br><br>This statement is required for submission and **will appear in the published article** if the submission is accepted. Please make sure it is accurate and that any funding sources listed in your Funding Information later in the submission form are also declared in your Financial Disclosure statement.<br><br>View published research articles from *PLOS ONE* for specific examples.<br><br>**NO authors have competing interests**<br><br>Enter: *The authors have declared that no competing interests exist.*<br><br>**Authors with competing interests**<br><br>Enter competing interest details beginning with this statement:<br><br>*I have read the journal's policy and the authors of this manuscript have the following competing interests: [insert competing interests here]*<br><br>* typeset	
**Ethics Statement**<br><br>Enter an ethics statement for this submission. This statement is required if the study involved:<br><br>• Human participants<br>• Human specimens or tissue<br>• Vertebrate animals or cephalopods<br>• Vertebrate embryos or tissues	N/A

• Field research<br><br>Write "N/A" if the submission does not require an ethics statement.<br><br>General guidance is provided below. Consult the submission guidelines for detailed instructions. **Make sure that all information entered here is included in the Methods section of the manuscript.**	

**Format for specific study types**<br><br>**Human Subject Research (involving human participants and/or tissue)**<br>• Give the name of the institutional review board or ethics committee that approved the study<br>• Include the approval number and/or a statement indicating approval of this research<br>• Indicate the form of consent obtained (written/oral) or the reason that consent was not obtained (e.g. the data were analyzed anonymously)<br><br>**Animal Research (involving vertebrate animals, embryos or tissues)**<br>• Provide the name of the Institutional Animal Care and Use Committee (IACUC) or other relevant ethics board that reviewed the study protocol, and indicate whether they approved this research or granted a formal waiver of ethical approval<br>• Include an approval number if one was obtained<br>• If the study involved *non-human primates*, add *additional details* about animal welfare and steps taken to ameliorate suffering<br>• If anesthesia, euthanasia, or any kind of animal sacrifice is part of the study, include briefly which substances and/or methods were applied<br><br>**Field Research**<br><br>Include the following details if this study involves the collection of plant, animal, or other materials from a natural setting:<br>• Field permit number<br>• Name of the institution or relevant body that granted permission	
**Data Availability**<br><br>Authors are required to make all data underlying the findings described fully available, without restriction, and from the time of publication. PLOS allows rare exceptions to address legal and ethical concerns. See the PLOS Data Policy and FAQ for detailed information.	Yes - all data are fully available without restriction

A Data Availability Statement describing where the data can be found is required at submission. Your answers to this question constitute the Data Availability Statement and **will be published in the article**, if accepted.<br><br>**Important:** Stating 'data available on request from the author' is not sufficient. If your data are only available upon request, select 'No' for the first question and explain your exceptional situation in the text box.<br><br>Do the authors confirm that all data underlying the findings described in their manuscript are fully available without restriction?	
**Describe where the data may be found in full sentences. If you are copying our sample text, replace any instances of XXX with the appropriate details.**<br><br>• If the data are **held or will be held in a public repository**, include URLs, accession numbers or DOIs. If this information will only be available after acceptance, indicate this by ticking the box below. For example: *All XXX files are available from the XXX database (accession number(s) XXX, XXX.*).<br>• If the data are all contained **within the manuscript and/or Supporting Information files**, enter the following: *All relevant data are within the manuscript and its Supporting Information files.*<br>• If neither of these applies but you are able to provide **details of access elsewhere**, with or without limitations, please do so. For example:<br><br>*Data cannot be shared publicly because of [XXX]. Data are available from the XXX Institutional Data Access / Ethics Committee (contact via XXX) for researchers who meet the criteria for access to confidential data.*<br><br>*The data underlying the results presented in the study are available from (include the name of the third party*	All relevant data are within the manuscript and its Supporting Information files.

and contact information or URL).<br>• This text is appropriate if the data are owned by a third party and authors do not have permission to share the data.<br><br>* typeset	
Additional data availability information:	

Powered by Editorial Manager® and ProduXion Manager® from Aries Systems Corporation

UNIVERSITY OF CALIFORNIA, LOS ANGELES

UCLA

BERKELEY · DAVIS · IRVINE · LOS ANGELES · RIVERSIDE · SAN DIEGO · SAN FRANCISCO

SANTA BARBARA · SANTA CRUZ

August 1, 2025

Dear Editors of PLOS One:

Please find our revised manuscript, "Increasing young people's use of sexual and reproductive health services in government health facilities in rural Kenya" for possible publication in PLOS One.

We believe that our original research paper will contribute meaningfully to the adolescent sexual and reproductive health (SRH) field in the Global South by describing a successful low-cost intervention that nearly doubled rural youth's use of SRH services in government facilities in less than one year.

We have modified the paper and title to no longer refer to "randomized controlled trial" because that did not describe properly the study design, which we have now referred to as a "randomized cluster study." We randomized facilities, not individuals, to different intervention types, and were not able to determine effects at the individual client level.

Lastly, we wish to confirm that:
-No co-author has competing interests to declare;
-Both co-authors have approved the manuscript for submission; and
-The content of the manuscript has not been published or submitted for publication elsewhere.

Thank you for your consideration.

Sincerely,

Paula Tavrow

Paula Tavrow, PhD
Adjunct Professor, Department of Community Health Sciences
UCLA Fielding School of Public Health
ptavrow@ucla.edu
(310) 435-1444

Manuscript

Click here to access/download;Manuscript;AHAP Paper Final _2025AUG1.docx

FULL TITLE

Increasing young people's use of sexual and reproductive health services in government health facilities in rural Kenya

SHORT TITLE

Increasing adolescent use of reproductive health services in Kenya

AUTHORS

Paula Tavrow*, ptavrow@ucla.edu, Department of Community Health Sciences, Fielding School of Public Health, University of California at Los Angeles, 650 Charles E. Young Dr. S, Los Angeles, CA 90095 USA

Krupa Maria Varghese, krupavarghese@g.ucla.edu, Department of Community Health Sciences, Fielding School of Public Health, University of California at Los Angeles, 650 Charles E. Young Dr. S, Los Angeles, CA 90095 USA

Albert Obbuyi, obbuyi@safaridoctors.org, Safari Doctors, P.O. Box 370-80500, Lamu, Kenya

Collins Juma, cjjuma@gmail.com, formerly of Centre for the Study of Adolescence, P.O. Box 19329-00202, Nairobi, Kenya

*Corresponding author.

ABSTRACT

Young people in sub-Saharan Africa, especially in rural areas, face numerous daunting geographic and psychosocial barriers to accessing sexual and reproductive health (SRH) services and information. Adolescents worry that providers will be judgmental and not accord them privacy and confidentiality. They are also concerned that community members or extended family members may see them waiting for SRH services, which could lead to being perceived as promiscuous and parental punishment. Because they lack comprehensive sexuality education (CSE) in schools, they have serious misconceptions about contraceptives. To address these barriers and increase youths' uptake of SRH services, the After-Hours Adolescent Project (AHAP) was created as low-cost intervention for rural government facilities. The main strategies involved extending clinic hours into early evening to be more convenient and confidential for youths, and training newly-graduated nurses to serve as AHAP nurses. AHAP was tested using a randomized cluster design in thirteen government health facilities in Western Kenya. The study examined two intervention types—the full intervention, which included having AHAP nurses conduct CSE classes in nearby schools and community locations, and a partial intervention, which was entirely clinic-based. After one year, AHAP increased young people's use of SRH services by 87% (full intervention 97%; partial intervention 77%), whereas comparison facilities saw no change. The full intervention facilities also attracted significantly more female and younger SRH clients. Additionally, improved nurse SRH attitudes after training were sustained. In sum, AHAP was acceptable and effective in rural settings, but requires ongoing budget outlays for nurse salaries.

KEYWORDS: adolescents, Kenya, sexual and reproductive health, rural, health facilities, access, adolescent and youth friendly services

BACKGROUND

It is estimated that young people aged 10-24 constitute close to a quarter of the world's population.(1) Among the world regions, sub-Saharan Africa has the largest percentage of young people, with about half of the population below the age of 18.(2) It also has significant sexual and reproductive health (SRH) challenges, particularly among the youth. The region accounts for the highest HIV burden globally, with HIV/AIDS being a leading cause of adolescent mortality, yet only about 25% of adolescents have ever been tested.(3,4) Sub-Saharan Africa also has the highest rate of adolescent pregnancies, with over 200 births per 1000 girls.(3) About 45% of adolescent pregnancies in the region are unintended and less than half of African girls report that their family planning needs were met.(5,6)

Like many other African countries, Kenya's young people have high levels of sexually transmitted disease and unwanted childbearing. Early sexual debut is common, with 47% of women and 55% of men in Kenya reporting initiation of sexual intercourse before age 18.(7) More than half of all new HIV infections occur among adolescents and youths.(7) With limited access to contraceptive services and legal abortions, Kenya's teenage pregnancy rate is 15%, which is associated with risky unsafe abortions, pre-eclampsia, birth complications, depression, and school dropouts.(8) In rural areas, girls' pregnancy rates are higher and they attempt to abort later, when it is more dangerous.(9) Only 10% of Kenyan students who give birth manage to return to school.(10) This contributes to a vicious cycle of poverty and high fertility, since early childbearing is associated with larger families, lower use of contraception, and fewer career options.

Barriers to adolescent access to information and services

Historically, sub-Saharan Africa has lagged in offering comprehensive SRH education and services to adolescents.(11) A dearth of comprehensive sexuality education in schools means that young

people generally lack accurate information about SRH and are often unaware that they can obtain family planning counseling and methods in health facilities without parental permission.(12,13) Providers' conservative values and attitudes also may limit the SRH information they give to young people. Moreover, internet access in rural areas is very limited, thereby preventing young people from accessing SRH information on their own. Societal taboos about discussing sex, inhibit conversations between adolescents and parents, teachers or clinicians.(12,14) Rural parents report feeling unequipped to discuss sexual health with their children.(15)

Concerning SRH services, many young people in Kenya and other African countries lack convenient access to appropriate health facilities, particularly in rural areas. Government health facilities have frequent contraceptive stock-outs, long waiting times, limited hours, and a lack of privacy and confidentiality.(12,16) Furthermore, conservative social norms and negative provider attitudes and behavior can deter young people from seeking SRH services.(17) Although Kenya's National Guidelines for Provision of Adolescent and Youth-Friendly Services (AYFS) emphasize the importance of catering to the specific needs of adolescents and youth, few health providers in rural areas have been trained to provide youth-friendly services.(18) Health providers sometimes refuse to offer contraceptives to unmarried or nulliparous adolescents due to perceptions that adolescents should be abstinent until marriage, that condoms will cause promiscuity, or that hormonal contraceptives will lead to infertility or other harms.(7,19,20)

Adolescents frequently report that providers are judgmental, uncaring and disrespectful.(12,19) A recent study found that healthcare providers' negative views about adolescent sexual activity could pose a major obstacle to girls' obtaining PrEP(21) Fear of receiving discouraging advice or judgment from providers, and general concerns about unfriendliness, have hindered youth uptake of SRH services.(22)

Relatedly, the older age of providers seems to impede youths' accessing of services. Adolescents in Kenya have reported feeling uncomfortable sharing their concerns with older adults.(12,22) One study found that young providers do have a positive effect on SRH service uptake.(22) Providers themselves sometimes report young clients as troublesome and suggest that these clients may feel more at ease with younger health workers.(21) Older providers describe language barriers because they do not understand the slang used by youths seeking SRH services. Younger providers who can connect with their clients and provide services in a friendly and empathetic way, rather than as parental figures, are generally believed to be more effective.(18)

Young people are also hesitant to access SRH services if they believe that their privacy and confidentiality will not be respected.(7,19,23) They fear being seen obtaining condoms or contraceptives due to social stigma and parental disapproval. They worry that providers, especially in rural areas, will pass on information about them to others.(12) Long waiting times and having to wait outdoors or in specified SRH areas also undermines confidentiality. Young people fear being spotted by relatives or acquaintances of their parents. Those with means sometimes travel to facilities further away to avoid being seen by those in their community.(7)

Health facilities' operating hours and distance pose other major challenges to young people's SRH use. Not only are service times inconvenient, but service providers are not always present during official hours or they close clinics early.(12,22) When health facilities are not open after school or on weekends, students are put in a difficult position of having to disclose health information to teachers to get permission to be excused from class.(7) In a study in South Africa, adolescents reported being unable to access services, because they were in school during clinic hours and were harassed by providers if they came late to a clinic.(24) Moreover, young people may have difficulty reaching facilities or affording SRH services. Adolescents in rural areas usually lack funds for

transport, consultation fees, pregnancy or STI tests, and supplies. Unless SRH services are free or very low cost, adolescents will have trouble accessing them.(7)

As a result of the barriers to obtaining SRH services at public health facilities, pharmacies have emerged as an alternative source of contraceptives.(25) Young people have reported preferring pharmacies over health facilities because they perceive them as more convenient and private.[23] They have considered pharmacy personnel to be less judgmental compared to facility staff. Additionally, when pharmacy personnel were similar in age to adolescents, they have felt more comfortable interacting with them.(26,27) However, pharmacies have also been shown to provide inadequate, and sometimes inaccurate, counselling on contraceptive use to adolescents.(25,27) Pharmacies also have a limited range of contraceptives and may not be affordable.(25) To reach all young people effectively with comprehensive SRH care and contraceptive services, it therefore continues to be important to increase access to adolescent-friendly government health facilities.

Youth-friendly service delivery standards and framework

About a decade ago, the WHO developed Global Standards for adolescent SRH that called on health facilities to improve adolescent health literacy, encourage community support for the utilization of services, offer an appropriate package of services, have competent and non-judgmental providers, be open during convenient hours with required infrastructure and supplies, offer services without discrimination, collect and use data, and involve adolescents in decision-making.(28) However, systematic reviews of interventions to improve SRH services for adolescents show that few have incorporated most or all of these standards.(29,30) One review noted that only interventions that integrate both demand and supply side activities could be considered ready for scaling up. Most interventions focused strictly on training staff or facility improvements, which did not have major impact.(31) In addition to gaps in intervention design, reviews have also called for rigorous evaluations of interventions to address the weak evidence base on effectiveness.(29,31)

To delineate the supply and demand-side factors that influence young people's use of SRH services, we previously had created a conceptual framework based on existing evidence.(32) From the supply-side, this framework depicted frontline health providers' attitudes and actions as paramount because they determine who receives services and under what conditions. Provider attitudes are influenced by their core beliefs, local values and norms, and empathy for clients. Their attitudes affect their actions, which can be modified through training and value clarification workshops, supervision, infrastructure, incentives, policies and regulations. From the demand-side, clients' use is influenced by their SRH knowledge, their felt need for service, convenience of services and costs (financial and psychosocial).(32) To improve access to SRH services in rural African government health facilities, it is vital to conduct rigorous trials to test the impact of manipulating these supply-side and demand-side factors.

Description of the intervention

Based on this conceptual framework, the Centre for the Study of Adolescence ([illegible]), with technical support from the UCLA Bixby Program, conducted a randomized cluster study to test the efficacy of the After-Hours Adolescent Project (AHAP). A[illegible]P aimed to increase adolescent clients' use of SRH services by building on existing resources available at rural government facilities. The two core elements were: (1) to keep health facilities open after hours, both to address the convenience factor and to reduce community members' knowledge of youths' use of services, and (2) to hire new young nurses to reduce the psychosocial costs associated with young people obtaining SRH services. The intervention aimed to improve providers' attitudes and actions by training them on youth-friendly services (YFS) and comprehensive sexuality education (CSE), ensuring adequate supplies of condoms at the facilities, and having monthly supervisor visits to problem-solve any difficulties.

The AHAP intervention components specifically sought to influence various aspects of the framework (see Fig. 1, black boxes). We hypothesized that adolescent use of SRH services would be strongly affected by providers' approachability and accessibility. At the same time, when more adolescents use the services, providers might be more inclined to see that young people have diverse needs and deserve individualized attention, which could in turn affect their attitudes. Supply-side factors tested by AHAP focused on the age of providers, years of practice, training on YFS and CSE, adequate infrastructure and supplies. Demand-side factors tested were convenience, privacy, better SRH knowledge through CSE, rapport with providers, and reduction of psychosocial costs.

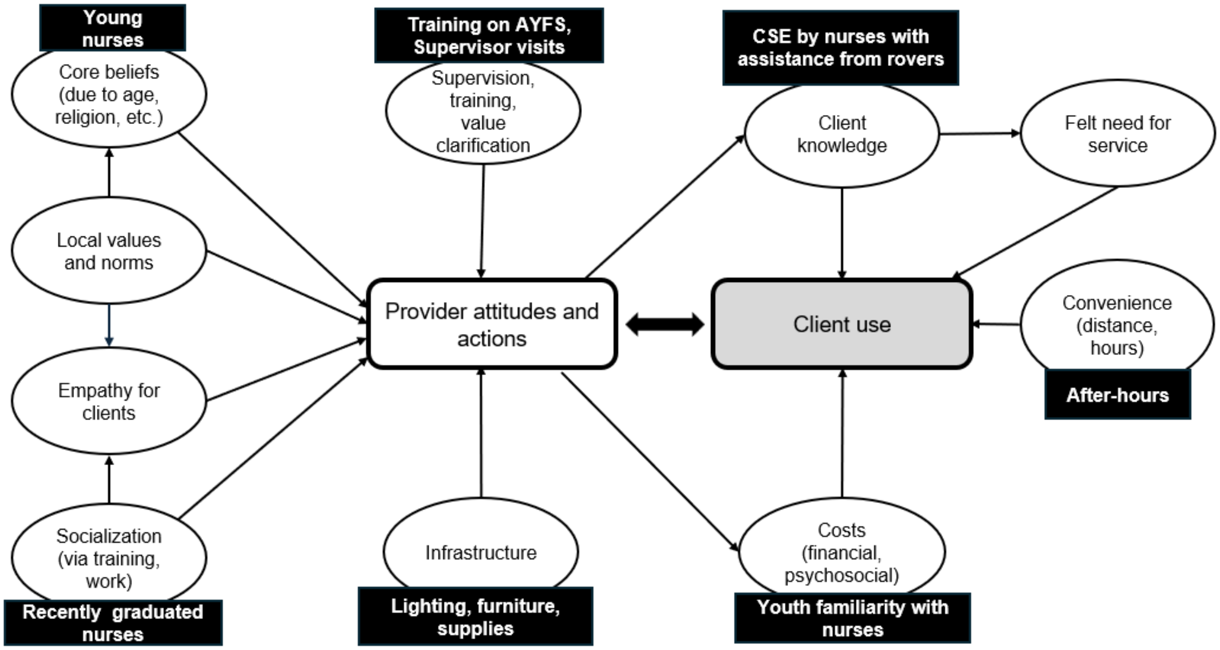

Fig 1: AHAP conceptual framework (black boxes show intervention components)

This study tested two versions of the program—the full intervention and a partial intervention. The full intervention differed from the partial intervention in that it included having the AHAP nurse conduct some CSE sessions in schools and the community. We wished to test if adding this element increased young people's familiarity with the nurse and overcame some psychological barriers to

access, as compared with the partial intervention which was entirely clinic-based. The full intervention thus was comprised of these five components: (1) convincing rural government health facilities to keep facilities open after normal hours (later on weekdays and for half-day on Saturdays); (2) recruiting one recent nursing school graduate to be posted in each participating health facility; (3) training the young nurses in YFS and CSE; (4) creating a cadre of rovers who provided logistics support to nurses conducting CSE in the community; and (6) improving facility infrastructure by adding lighting (if necessary), providing board games and informational brochures, and ensuring adequate availability of condoms. While the partial intervention did not include nurse-led CSE in the community, it did encompass all the other components.

In the full intervention, the nurses sometimes conducted CSE in the late morning and arrived in the afternoon at the clinics, remaining there until dark. Each nurse visited 4-5 schools in their catchment area, which included both primary and secondary schools for both boys and girls. The nurses were assisted by a new cadre of rovers set up by the program. Rovers were young high school graduates who were in their waiting period between completing their high school examinations and possibly joining a college. These rovers helped nurses with the logistics of setting up the CSE classes at schools to make the process more efficient. They also helped the AHAP nurses occasionally conduct CSE on the weekend or in community centers to reach out-of-school youths and young couples. In contrast, for the partial intervention, nurses worked a staggered schedule in the facilities (generally from 10:30 AM to 6:30 PM) and did no community outreach. In both intervention types, AHAP nurses also worked one half-day on the weekend. Special clinic registers were provided to record visits that took place during the extended hours.

Ten new nursing graduates (23-29 years old) were recruited specifically for the provision of SRH services to adolescents at the intervention facilities. Nurses underwent a 3-day training with role plays, values clarification, and discussions on adolescent friendly services to ensure their alignment

with the program. Specifically, AHAP trained nurses on the importance of ensuring equitable, accessible, acceptable, appropriate, and effective care. They also received a 5-day training on how to provide adolescents with CSE and dispel SRH myths. The curriculum included information on puberty, abstinence, consent, contraception, sexually-transmitted infections, peer pressure, sexual harassment and rape, and young people's rights to SRH services. None of the nurses had previously received CSE.

Lastly, AHAP carried out basic facility-level infrastructure and supply improvements. Given low electricity availability, solar panels were installed at some facilities so that services could be provided during twilight and even after dark. The facilities were also made more attractive to the youth by setting up basic adolescent corners with board games and information brochures on SRH. Chairs and tables were arranged to provide privacy during consultation, and supply cabinets for condoms and registers were purchased. Anonymous client cards and lockboxes were also provided so that youth clients could give their confidential feedback on services received.

METHODS

The purpose of the study was to test whether making SRH services more convenient, private, confidential, and youth-friendly increased young people's use of rural government health facilities. The team approached government health facilities in a rural county of Western Kenya to invite them to participate in the study. Of fifteen facilities approached, thirteen agreed to participate. Through a randomization process, five facilities were assigned to receive the full intervention, four were assigned to the partial intervention, and four were not assigned to any intervention (control facilities).

The study was conducted between April 2018 and June 2019. Baseline data were collected from all facility registers for the month of April 2018 before the program began, and included information

on clinic opening hours and number of young people visiting for SRH and non-SRH reasons. Recent nursing graduates were recruited as AHAP nurses in May 2018 and a baseline survey of their knowledge, attitudes, and perceptions of adolescent sexual health and services was collected prior to training. After the program was operational for 10 months, endline data was collected from facility registers for the month of April 2019, following the same parameters as the baseline. Nurses were also re-surveyed.

Both the full and partial intervention facilities were also provided with feedback cards for clients which allowed young people to share their satisfaction with the services provided and give suggestions for improvement. Feedback cards were collected from July 2018 to June 2019. The cards included data on client age, gender, facility-visited, reason for visit, provider's gender, and questions about services which covered privacy, availability of supplies and quality of interaction with the provider. Additionally, one focus group discussion was conducted with AHAP nurses and another with youths in a facility catchment area to capture their experience with the program.

Pearson's χ^2 and F-tests were used to compare across groups as appropriate, with $p<0.05$ considered statistically significant. Data was analyzed using SPSS version 30. Regarding ethical approval, the University of California, Los Angeles Institutional Review Board determined that the AHAP study was exempted because it was a program review of secondary data.

RESULTS

The key finding of this study was that facilities with AHAP nurses significantly increased the number of SRH service visits by young people, with the greatest change in the full intervention facilities. At baseline, the thirteen facilities were serving a catchment of nearly 105,000 people of all ages, with 144 clinic staff (see Table 1). Monthly youth SRH visits in the AHAP facilities at baseline in April 2018 totaled 1,031, which rose to 1,931 in April 2019 after ten months of the

project, representing an 87.3% increase. Meanwhile, the control facilities as a whole had 337 monthly youth SRH visits at baseline and 337 at endline, which indicated that there had been no change. The full intervention facilities registered overall a 96.8% visit increase even though one of the facilities lost their AHAP provider in the month before the endline assessment, which was significantly higher than the 77.2% increase in the partial intervention facilities.

The study also found that 52.2% of the SRH visits in April 2019 occurred after hours in the full intervention facilities, and 37.6% occurred after hours in the partial intervention facilities, which suggested that the increase in youth SRH visits arose mainly during times when facilities generally were closed. The registers also showed that AHAP nurses usually kept the facilities open until 6 or 7 PM on weekdays, although one facility closed at 5 PM and one hospital stayed open until 8 PM. Moreover, most facilities did offer some Saturday hours, although two never opened on the weekends.

Table 1: SRH visits by adolescents in full-intervention, partial intervention and control facilities, before and after intervention

Facility	Catchment population	No. of staff	No. of SRH youth visits (April 2018)	No. of SRH youth visits (April 2019)	Change over time (%)	Came after hours (%)	Weekday hours of AHAP nurse	Saturday hours of AHAP nurse
**Full AHAP**								
Facility F1*	18,177	23	272	254	-6.6	57.3	11-7 PM	12-6 PM
Facility F2	4,378	3	13	122	838.5	41.4	10-6 PM	None
Facility F3	4,614	2	47	201	327.7	71.2	10-6 PM	10-6 PM
Facility F4	4,903	4	163	299	83.4	66.2	11-6 PM	11-6 PM
Facility F5	11,585	7	37	171	362.2	24.9	9-7 PM	9 -7 PM
**Subtotal Full**	**43,657**	**39**	**532**	**1047**	**96.8**	**52.2**		
**Partial AHAP**								
Facility P1	2,722	6	51	160	213.7	56.1	10-10 PM	8-10 AM
Facility P2	5,938	2	91	146	60.4	17.4	9 -5 PM	10-4 PM

Facility P3	2,412	3	122	219	79.5	19.5	9:30 -6 PM	None
Facility P4	26,239	75	235	359	52.8	57.4	11- 8 PM	11-8 PM
**Subtotal Partial**	**37,311**	**86**	**499**	**884**	**77.2**	**37.6**		
**Control Facilities**								
Facility C1	5,172	4	65	87	33.8	---	None	None
Facility C2	3,783	3	47	49	4.3	---	None	None
Facility C3	5,118	3	74	53	-28.4	---	None	None
Facility C4	9,958	9	151	148	-2.0	---	None	None
**Subtotal Control**	**24,031**	**19**	**337**	**337**	**0**			

* Note: AHAP provider left in early March 2019 prior to the endline assessment in April 2019. The nurse was not replaced.

The study also assessed nurse attitudes at baseline and endline about family planning methods, sexual norms, and HIV/AIDS issues. Of the ten nurses who were trained at baseline, seven took the survey. At follow-up, these seven nurses and one new nurse (who had replaced an AHAP nurse midway through the project) took the survey. Of the twelve attitudes assessed, the study found that nurse attitudes shifted significantly in a more "youth-friendly" direction for six of the attitudes, of which most of them related to family planning (see Table 2). The remaining six attitudes measured also shifted in the desired direction, but the change was not significant. Notably, none of the nurses still believed that contraceptives were harmful to adolescents, that condoms had holes in them, or that young people needed parental permission to obtain family planning.

Table 2. AHAP nurse attitudes and beliefs, at baseline and follow-up (9 months later)

Statement	Baseline (n=7)	Follow-up (n=8)	F	p-value
**Family Planning**				
Some family planning methods cause cancer.	2.43	1.25	6.01	**0.029**
If a girl uses family planning methods for long, she can become infertile.	2.57	1.38	5.99	**0.029**
People who are 13 years of age are mature enough to make decisions regarding sexual activity.	1.57	4.00	37.11	**0.000**

All people should have the right to choose what happens to their own body.	3.71	4.88	6.35	**0.026**
If an adolescent is requesting family planning, parents should be notified first.	2.14	1.13	4.29	0.059
**Sexual Norms**				
Someone who has several sexual partners is a slut.	3.14	1.38	14.13	**0.002**
When a girl had been sexually assaulted, she usually had provoked it.	1.43	1.25	0.30	0.595
Young people should abstain from sex before marriage.	4.00	2.50	6.07	**0.029**
About half of rape accusations are false.	2.43	2.00	0.76	0.399
**HIV/AIDS**				
It is not a guarantee that a person will get HIV if he/she has unprotected sex with an HIV positive person.	2.86	3.63	1.07	0.320
Some condoms have pores in them.	2.43	1.50	2.66	0.127
HIV can be transmitted through kissing.	2.14	1.38	2.25	0.158

Notes: Answers based on a 5-point scale: (1) Strongly disagree; (2) Disagree; (3) Unsure; (4) Agree; and (5) Strongly agree. Significant p-values are in bold.

Client feedback cards were also assessed by the study team. A total of 1954 cards were retrieved from locked boxes in all AHAP facilities, of which 1050 were from full intervention facilities and 904 in partial intervention facilities. The study found that about three-quarters of the cards were filled in by youths under age 21, with younger youths aged 10-17 significantly more likely to be in the full AHAP facilities (see Table 3). Females were more likely than males to fill out cards (55% to 45%), but there were significantly more females (61%) at full intervention facilities than partial intervention facilities (48%). The SRH services that young people desired were mainly condoms (31%), family planning (27%) or testing for HIV or sexually-transmitted diseases (26%). Young people were more likely to come for family planning services in the full intervention facilities than the partial facilities. Counselling only was desired by about 8% of those who filled in the cards.

Overall, client satisfaction seemed to be very high, with 96-99% of the youths who filled in the cards reporting that they received the information and services they wanted, and that they were comfortable coming for services, had enough privacy, and the nurse was not harsh to them. For

some of these items, satisfaction with the full intervention was higher than for the partial intervention. Waiting times overall were very low (almost always less than 15 minutes).

Because the full intervention had more young people reporting that they were seen by a female provider, the study assessed whether having a female provider was associated with greater satisfaction or higher use of family planning services. However, the gender of the provider was not found to be a significant factor (data not shown).

Table 3. Comparison of full AHAP versus partial AHAP intervention, by client satisfaction cards

		**Intervention type**				**Total**		**Chi-sq**	**p-value**
		**Full AHAP**		**Partial AHAP**					
**Age (years)**	10-17	319	(30.4%)	168	(18.6%)	487	(24.9%)		
	18-21	512	(48.8%)	444	(49.1%)	956	(48.9%)		
	22-30	219	(20.9%)	292	(32.3%)	511	(26.2%)		
	**Total**	**1050**	**(100.0%)**	**904**	**(100.0%)**	**1954**	**(100.0%)**	**51.46**	**0.000**
**Client gender**	Male	401	(39.3%)	465	(51.6%)	866	(45.0%)		
	Female	620	(60.7%)	437	(48.4%)	1057	(55.0%)		
	**Total**	**1021**	**(100.0%)**	**902**	**(100.0%)**	**1923**	**(100.0%)**	**29.16**	**0.000**
**Main service desired**	Family planning	321	(31.4%)	206	(22.9%)	527	(27.4%)		
	Pregnancy test or ANC/PNC	56	(5.5%)	61	(6.8%)	117	(6.1%)		
	Condoms	300	(29.4%)	299	(33.2%)	599	(31.2%)		
	VCT/STI	281	(27.5%)	209	(23.2%)	490	(25.5%)		
	Counseling only	38	(3.7%)	120	(13.3%)	158	(8.2%)		
	Other	25	(2.4%)	6	(0.7%)	31	(1.6%)		
	**Total**	**1021**	**(100.0%)**	**901**	**(100.0%)**	**1922**	**(100.0%)**	**82.92**	**0.000**
**Provider gender**	Male nurse	680	(63.8%)	734	(79.6%)	1414	(71.2%)		
	Female nurse	385	(36.2%)	188	(20.4%)	573	(28.8%)		
	**Total**	**1065**	**(100.0%)**	**922**	**(100.0%)**	**1987**	**100.0%)**	**59.81**	**0.000**
**Waiting time**	0-5 minutes	509	(51.3%)	489	(63.7%)	998	(56.7%)		
	6-15 minutes	454	(45.7%)	183	(23.8%)	637	(36.2%)		

	16+ minutes	30	(3.0%)	96	(12.5%)	126	(7.2%)		
	**Total**	**993**	**100.0%)**	**768**	**(100.0%)**	**1761**	**(100.0%)**	**123.5**	**0.000**
**Opinions about services**	Comfortable talking to provider	1039	(99.7%)	902	(99.0%)	1941	(99.4%)	**6.37**	**0.041**
	Obtained Information wanted	1036	(99.7%)	904	(99.2%)	1940	(99.5%)	2.19	0.139
	Obtained supplies or tests wanted*	1031	(99.8%)	899	(97.7%)	1930	(99.3%)	**16.60**	**0.000**
	Provider was not harsh	1022	(98.3%)	837	(93.1%)	1859	(95.9%)	**37.958**	**0.000**
	Had enough privacy	1023	(98.6%)	881	(97.8%)	1904	(98.2%)	4.133	0.127
	Would be comfortable coming again	1034	(99.5%)	888	(98.7%)	1922	(99.1%)	**8.168**	**0.017**

Notes: *Includes those who didn't want anything. ANC/PNC= antenatal care/postnatal care. VCT/STI= voluntary counselling & testing/sexually-transmitted infections.

Lastly, in a focus group, adolescents said that they considered AHAP nurses to be very friendly. They found it empowering to be able to ask for AHAP nurses by name when they visited the facility for SRH services. If the AHAP nurse was not present, adolescents reported leaving without being seen to avoid being embarrassed by other health providers. They appreciated the educative and nonjudgmental approach of AHAP nurses, as well as the privacy and confidentiality of the AHAP services in the late afternoon when the facilities had no other clients.

AHAP nurses in their focus group described that they liked how AHAP brought together schools and health facilities through the CSE instruction in the full intervention. They felt privacy and trust were the most important issues for young people, and that providing CSE in the community helped adolescents overcome their shyness. The nurses stressed that "information is power" and CSE helped young people make sound decisions. Adolescents told them that their teachers were biased, and they appreciated having a nurse to whom they could talk openly about their SRH concerns. The nurses also noted that some school principals at elementary schools were pleased that during

AHAP no pregnancies among school girls had occurred. The nurses were also able to effectively deal with any community concerns about the program. During a dissemination workshop in the district, one facility in-charge explained:

> *Youths themselves are very particular. If it is an AHAP nurse, they come. If it is someone else, they won't come. At first, we had some challenges because so many young people were coming after hours. One school principal wanted to know why students are there [at the facility] at 5-6 PM. We held a stake-holders meeting and sensitized the community [about AHAP]. After that, we had no problems.*

DISCUSSION

This study used a randomized cluster design to evaluate the effectiveness of a system improvement intervention to increase adolescent uptake of SRH services at rural government health facilities in Kenya. The two core strategies were to extend the hours of services into the early evening and a few hours on the weekend, and to employ newly-graduated nurses who were trained in YFS and CSE. Our evaluation found that in facilities that implemented the full AHAP intervention, the number of young people utilizing SRH services almost doubled (increase of 96.8%) as compared to no change at all in the control facilities. In facilities that implemented the partial AHAP (without the community engagement component), the number of young SRH clients still increased by 77%.

Extending facility hours appeared to be a critical component of the intervention, since half of the young people in the full intervention and one-third in the partial intervention accessed services outside of the normal operating hours. Other studies have noted that inflexible and inconvenient opening times of facilities are a major access barrier to adolescent's use of SRH services, with regular facility hours overlapping with school hours, resulting in risks to students' privacy by having to obtain their teacher's permission.(7,12) Additionally, excessive waiting times and

frequent absences of staff discourage young people. Our findings align with previous research that had indicated that convenient facility hours improved adolescent use of HIV testing, family planning methods, and STI management in Africa.(33,34)

Another barrier that AHAP addressed was the dearth of SRH providers in rural facilities with whom young people feel comfortable consulting. Fear of judgement from older providers, who may be perceived as parental figures, makes it difficult for adolescents to communicate their needs.(18) Moreover, researchers have found that long-term socialization into existing health systems can result in reduction in empathy towards patients.(32) AHAP's hiring of recently-graduated young nurses meant that adolescents were meeting with nurses closer to their age who had not become inured to the problems faced by youths. Regarding adolescents' preferences for younger providers, previous research was mixed. Some studies suggested that younger providers are preferred as they can better understand the SRH challenges of youth; however, other studies indicated a preference for older providers because they were perceived to be more competent or experienced.(35–37) We hypothesize that AHAP's intensive training ensured that staff were competent even though they were young, which meant that they met both preferences identified in previous studies.

The AHAP training and implementation seemed to re-align nurses' attitudes towards family planning services for young people, and other progressive norms. Even though the AHAP nurses were recent graduates, at the onset of the program they held some inaccurate and problematic beliefs about SRH care for adolescents, as seen in their baseline attitudinal scores. The AHAP training and their subsequent interactions with young people appeared to result in a sustained improvement in nurses' attitudes and beliefs, as evidenced by their scores at endline. More providers recognized that adolescents had the right to services without parental consent, and fewer believed negative myths about health effects of family planning methods or the unreliability of condoms. These results were in line with previous studies in Kenya, Malawi and Nigeria, which

head found that capacity-building and supportive supervision of health providers improved their attitudes, such as recognizing their adolescents' rights to obtain contraceptives.(31,34,38,39)

In comparing the full to the partial intervention, we found significant differences in uptake of SRH services, types of services availed by adolescents, and the gender/age composition of the clients. Adolescents attending the full intervention facilities were more likely to request family planning and VCT/STI services. The full intervention facilities also seemed to attract significantly more female and younger clients. These results have parallels in previous studies, which had found that a combination of facility improvement and community outreach was more effective than a single-component interventions.? Having school-based and community-based CSE sessions led by an AHAP nurse from a nearby facility may have overcome the anxieties of female and younger youths, who are more likely to be deterred from using SRH services due to worries about nurse shaming and parental punishment. Previous studies in Zambia and Kenya have also found that integrated CSE by trained providers can result in better SRH outcomes amongst adolescents, including reduction in adolescent pregnancy.(40,41) We hypothesize that a key factor influencing the effectiveness of the CSE component was the role of the rovers. This cadre allowed nurses not to have to spend extra time in the planning and logistics of offering CSE classes, making the process more efficient and feasible.

While the increase in adolescent uptake of SRH services was an intended outcome, our implementation also revealed some unintended benefits of the program that the study did not measure. The program seemed to have had high levels of acceptability amongst multiple stakeholders. The addition of young, recently graduated nurses provided facilities with more human resources. Although most facilities could not retain these staff post program because of budgetary constraints, during the course of the intervention period AHAP nurses supported facility staff in their tasks beyond adolescent care and ensured that the facility stayed open. We also heard

anecdotally that female drop-outs due to pregnancy in nearby schools had reduced, resulting in parents and teachers being supportive of the program.

Our study had several limitations. First, it was conducted in only 13 facilities in one county in Western Kenya, so it may not be widely generalizable. However, the sample included a range of rural government facilities--from dispensaries and health centers to district hospitals--which suggests that AHAP could be effective across the spectrum of public facilities. Another limitation was that the study did not assess the difference in costs between the full and partial interventions. Even though the full intervention significantly increased uptake as compared to the partial intervention (97 % vs 77%), it is possible that the added expense and complexity of the community-level CSE activities would not be feasible for some localities. A final limitation relates to the intervention itself. Although AHAP was highly acceptable and achieved dramatic improvements in service use, the facilities did not retain AHAP nurses after the conclusion of the program because they had not included their salaries in their annual budgets. It would be important to ensure at the onset that facilities would be prepared to absorb the cost of an additional salary at the conclusion of the program.

Our study also had several strengths. First, our randomized cluster design added validity and rigor to our findings, which may not be the case with other similar studies and responded to the call for more experimental studies on intervention effectiveness. Second, our evaluation used multiple methods to provide insights on the impact of the intervention on the uptake of SRH services among adolescents, but also on changes in types of services availed, patient satisfaction and attitudes of AHAP providers. This permitted a more comprehensive assessment of the intervention's effectiveness. Lastly, AHAP relied on existing government health facilities and schools, which meant that the program was grounded in the local context and responded to the local challenges.

CONCLUSION

This randomized cluster study demonstrated that it was possible to nearly double significantly young people's use of government health facilities for SRH services in rural Kenya. AHAP directly addressed some of the most entrenched psychosocial barriers facing young rural Kenyans. It allowed adolescents to obtain SRH services confidentially and privately, ensured that free condoms were always available, and put adolescents on more equal footing with young providers who were disinclined to be harsh or judgmental. Having AHAP nurses deliver CSE in schools and the community in the full intervention further helped to increase adolescents' familiarity with them and showed that they were not biased against young people. This combination of factors, not any single item, enabled a low-cost activity, relying entirely on public health facilities, to increase significantly adolescent SRH services use.

ACKNOWLEDGEMENTS

We are grateful for funding provided by the Lalor Foundation and Save the Children (Sweden).

References:

1. World Population Dashboard [Internet]. [cited 2024 Aug 7]. Available from: https://www.unfpa.org/data/world-population-dashboard

2. Population Trends in Africa [Internet]. Population Reference Bureau. [cited 2024 Aug 21]. Available from: https://2023-wpds.prb.org/africa/

3. Shayo FK, Kalomo MH. Prevalence and correlates of sexual intercourse among sexually active in-school adolescents: an analysis of five sub-Sahara African countries for the adolescent's sexual health policy implications. BMC Public Health. 2019 Oct 12;19(1):1285.

4. Hlophe LD, Tamuzi JL, Shumba CS, Nyasulu PS. Barriers and facilitators to anti-retroviral therapy adherence among adolescents aged 10 to 19 years living with HIV in sub-Saharan Africa: A mixed-methods systematic review and meta-analysis. PLoS One. 2023 May 18;18(5):e0276411.

5. Harrington EK, Casmir E, Kithao P, Kinuthia J, John-Stewart G, Drake AL, et al. "Spoiled" girls: Understanding social influences on adolescent contraceptive decision-making in Kenya. Ortega JA, editor. PLoS ONE. 2021 Aug 12;16(8):e0255954.

6. Chandra-Mouli V, Akwara E. Improving access to and use of contraception by adolescents: What progress has been made, what lessons have been learnt, and what are the implications for action? Best Practice & Research Clinical Obstetrics & Gynaecology. 2020 Jul;66:107–18.

7. Mutea L, Ontiri S, Kadiri F, Michielesen K, Gichangi P. Access to information and use of adolescent sexual reproductive health services: Qualitative exploration of barriers and facilitators in Kisumu and Kakamega, Kenya. Baysari MT, editor. PLoS ONE. 2020 Nov 12;15(11):e0241985.

8. Mutea L, Ontiri S, Macharia S, Tzobotaro M, Ajema C, Odiara V, et al. Evaluating the effectiveness of a combined approach to improve utilization of adolescent sexual reproductive health services in Kenya: a quasi-experimental design study protocol. Reprod Health. 2019 Dec;16(1):153.

9. Ushie BA, Izugbara CO, Mutua MM, Kabiru CW. Timing of abortion among adolescent and young women presenting for post-abortion care in Kenya: a cross-sectional analysis of nationally-representative data. BMC Women's Health. 2018 Dec;18(1):41.

10. Undie CC, Birungi H, Odwe G, Obare F. Expanding access to secondary school education for teenage mothers in Kenya: A baseline study report. Reproductive Health [Internet]. 2015 Jan 1; Available from: https://knowledgecommons.popcouncil.org/departments_sbsr-rh/256

11. Ross DA, Mshana G, Guthold R. Adolescent Health Series: The health of adolescents in sub-Saharan Africa: Challenges and opportunities. Tropical Medicine & International Health. 2021;26(11):1326–32.

12. Newton-Levinson A, Leichliter JS, Chandra-Mouli V. Sexually Transmitted Infection Services for Adolescents and Youth in Low- and Middle-Income Countries: Perceived and Experienced Barriers to Accessing Care. Journal of Adolescent Health. 2016 Jul;59(1):7–16.

13. Ninsiima LR, Chiumia IK, Ndejjo R. Factors influencing access to and utilisation of youth-friendly sexual and reproductive health services in sub-Saharan Africa: a systematic review. Reprod Health. 2021 Dec;18(1):135.

14. Miller LE, Zamudio-Haas S, Otieno B, Amboka S, Odeny D, Agot I, et al. "We Don't Fear HIV. We Just Fear Walking around Pregnant.": A Qualitative Analysis of Adolescent Sexuality and Pregnancy Stigma in Informal Settlements in Kisumu, Kenya. Studies in Family Planning. 2021 Dec;52(4):557–70.

15. Ijeoma U. Parent–adolescent communication on adolescent sexual and reproductive health in sub-Saharan Africa: a qualitative review and thematic synthesis. 2021;

16. Capurchande R, Coene G, Schockaert I, Macia M, Meulemans H. "It is challenging… oh, nobody likes it!": a qualitative study exploring Mozambican adolescents and young adults' experiences with contraception. BMC Women's Health. 2016 Dec;16(1):48.

17. Melesse DY, Mutua MK, Choudhury A, Wado YD, Faye CM, Neal S, et al. Adolescent sexual and reproductive health in sub-Saharan Africa: who is left behind? BMJ Global Health.

18. Godia PM, Olenja JM, Lavussa JA, Quinney D, Hofman JJ, van den Broek N. Sexual reproductive health service provision to young people in Kenya; health service providers' experiences. BMC Health Services Research. 2013 Nov 14;13(1):476.

19. Ormel H, Oele G, Kok M, Oruko H, Oluoch B, Smet E, et al. Reducing unmet need for contraceptive services among youth in Homabay and Narok counties, Kenya: the role of community health volunteers – a qualitative study. BMC Health Serv Res. 2021 Dec;21(1):405.

20. Tavrow P, Karei EM, Obbuyi A, Omollo V. Community Norms About Youth Condom Use in Western Kenya: Is Transition Occurring? 2012;

21. Lanham M, Ridgeway K, Mireku M, Nhamo D, Pillay D, Murire M, et al. Health care providers' attitudes toward and experiences delivering oral PrEP to adolescent girls and young women in Kenya, South Africa, and Zimbabwe. BMC Health Serv Res. 2021 Dec;21(1):1112.

22. Godia PM, Olenja JM, Hofman JJ, Van Den Broek N. Young people's perception of sexual and reproductive health services in Kenya. BMC Health Serv Res. 2014 Dec;14(1):172.

23. Aninanya GA, Debpuur CY, Awine T, Williams JE, Hodgson A, Howard N. Effects of an Adolescent Sexual and Reproductive Health Intervention on Health Service Usage by Young People in Northern Ghana: A Community-Randomised Trial. Zeeb H, editor. PLoS ONE. 2015 Apr 30;10(4):e0125267.

24. Mokomane Z, Mokhele T, Mathews C, Makoae M. Availability and accessibility of public health services for adolescents and young people in South Africa. Children and Youth Services Review. 2017 Mar;74:125–32.

25. Gonsalves L, Kamuyango A, Chandra-Mouli V. Pharmacies: an important source of contraception for some adolescents, but not a panacea for all. Sexual and Reproductive Health Matters. 2023 Dec 31;31(1):2221883.

26. Gonsalves L, Wyss K, Cresswell JA, Waithaka M, Gichangi P, Martin Hilber A. Mixed-methods study on pharmacies as contraception providers to Kenyan young people: who uses them and why? BMJ Open. 2020 Jul;10(7):e034769.

27. Gonsalves L, Wyss K, Gichangi P, Hilber AM. Pharmacists as youth-friendly service providers: documenting condom and emergency contraception dispensing in Kenya. Int J Public Health. 2020 May;65(4):487–96.

28. Nair M, Baltag V, Bose K, Boschi-Pinto C, Lambrechts T, Mathai M. Improving the Quality of Health Care Services for Adolescents, Globally: A Standards-Driven Approach. J Adolesc Health. 2015 Sep;57(3):288–98.

29. Gottschalk LB, Ortayli N. Interventions to improve adolescents' contraceptive behaviors in low- and middle-income countries: a review of the evidence base. Contraception. 2014 Sep;90(3):211–25.

30. Nash K, O'Malley G, Geoffroy E, Schell E, Bvumbwe A, Denno DM. "Our girls need to see a path to the future" --perspectives on sexual and reproductive health information among adolescent girls, guardians, and initiation counselors in Mulanje district, Malawi. Reprod Health. 2019 Jan 25;16(1):8.

31. Denno DM, Plesons M, Chandra-Mouli V. Effective strategies to improve health worker performance in delivering adolescent-friendly sexual and reproductive health services. International Journal of Adolescent Medicine and Health. 2021 Dec 1;33(6):269–97.

32. Tavrow P. Promote or discourage: how providers can influence service use. In: Social determinants of sexual and reproductive health: informing future research and programme implementation/edited by Shawn Malarcher. 2010. (15).

33. Kose J, Tiam A, Ochuka B, Okoth E, Sunguti J, Waweru M, et al. Impact of a Comprehensive Adolescent-Focused Case Finding Intervention on Uptake of HIV Testing and Linkage to Care Among Adolescents in Western Kenya. JAIDS Journal of Acquired Immune Deficiency Syndromes. 2018 Nov 1;79(3):367.

34. Rosenberg NE, Bhushan NL, Vansia D, Phanga T, Maseko B, Nthani T, et al. Comparing Youth-Friendly Health Services to the Standard of Care Through "Girl Power-Malawi": A Quasi-Experimental Cohort Study. JAIDS Journal of Acquired Immune Deficiency Syndromes. 2018 Dec 1;79(4):458.

35. Uka VK, White H, Smith DM. The sexual and reproductive health needs and preferences of youths in sub-Saharan Africa: A meta-synthesis. PLOS ONE. 2024 Dec 31;19(12):e0300829.

36. Geary RS, Webb EL, Clarke L, Norris SA. Evaluating youth-friendly health services: young people's perspectives from a simulated client study in urban South Africa. Global Health Action. 2015 Dec 1;8(1):26080.

37. Arije O, Hlungwani T, Madan J. "They will judge you more like a parent instead of like a health practitioner": Perceptions and preferences of young people regarding sexual and reproductive health services in Southwest Nigeria. Dialogues in Health. 2022 Dec 1;1:100051.

38. Agu IC, Agu C, Mbachu C, Onwujekwe O. Impact of a capacity-building intervention on views and perceptions of healthcare providers towards the provision of adolescent sexual and reproductive health services in southeast Nigeria: a cross-sectional qualitative study. BMJ Open. 2023 Nov 24;13(11):e073586.

39. Okinyi HM, Wachira CM, Wilson KS, Nduati MN, Onyango AD, Mburu CW, et al. "I Have Actually not Lost any Adolescent Since I Started Engaging Them one on one:" Training Satisfaction and Subsequent Practice among Health Providers Participating in a Standardized Patient Actor Training to Improve Adolescent Engagement in HIV Care. J Int Assoc Provid AIDS Care. 2022 Jan 1;21:23259582221075133.

40. Mbizvo MT, Kasonda K, Muntalima NC, Rosen JG, Inambwae S, Namukonda ES, et al. Comprehensive sexuality education linked to sexual and reproductive health services reduces early and unintended pregnancies among in-school adolescent girls in Zambia. BMC Public Health. 2023 Feb 16;23(1):348.

41. Ogolla MA, Ondia M. Assessment of the Implementation of Comprehensive Sexuality Education in Kenya. Afr J Reprod Health. 2019 Jun;23(2):110–20.

Figure 1

Click here to access/download;Figure;Fig1.tif

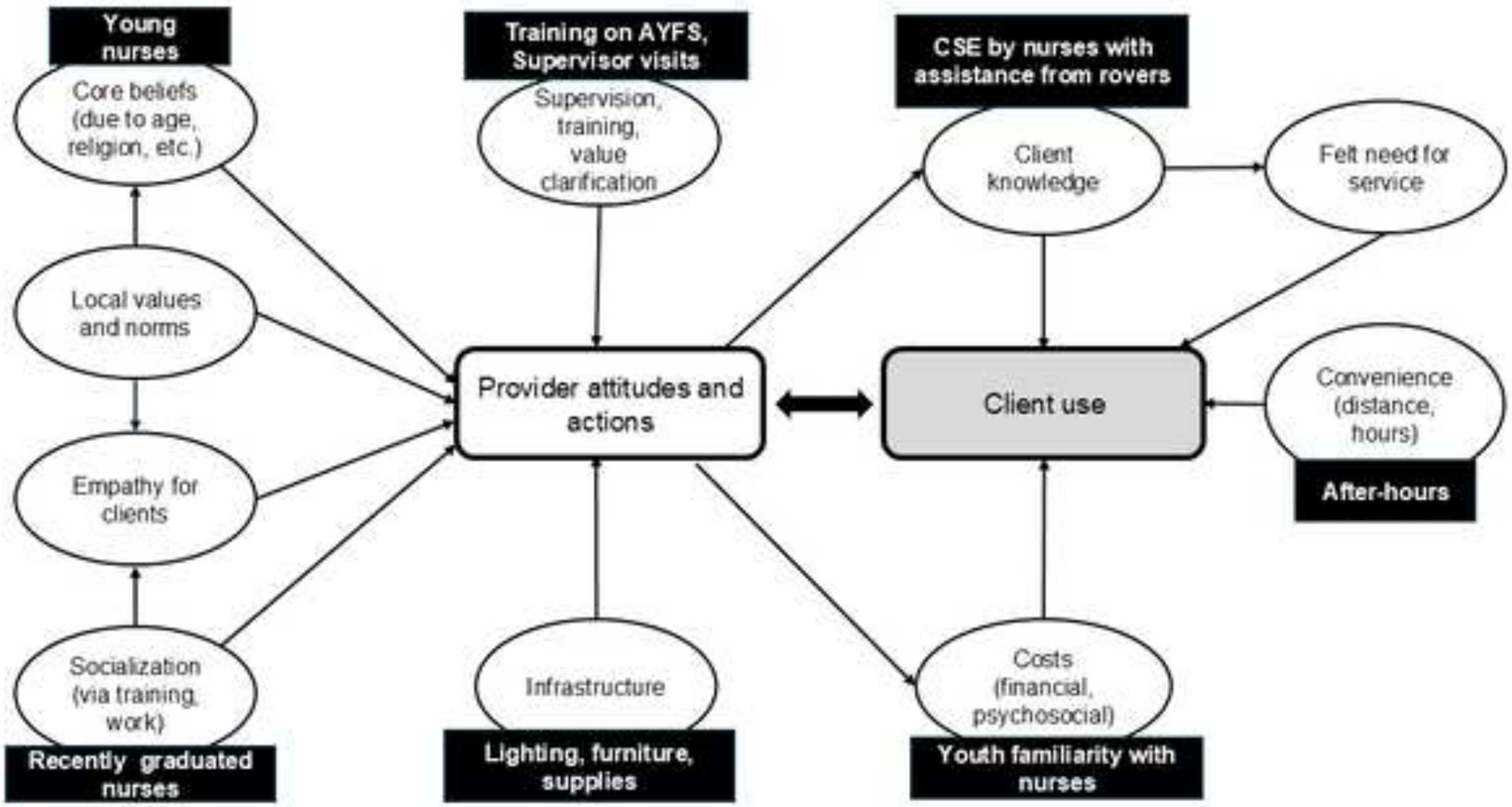

Data files zip

Click here to access/download

Supporting Information - Compressed/ZIP File Archive

AHAP RCT Data files.zip
