# Supplementary material for: Increasing young people’s use of sexual and reproductive health services in government health facilities in rural Kenya
Source: PLoS One. 2026 May 4;21(5):e0347329. doi: 10.1371/journal.pone.0347329 (PMC13138647; doi:10.1371/journal.pone.0347329)
Supplement: Attachment — Submitted filename: Response to Reviewers.docx [file pone.0347329.s006.docx]

Nov. 3, 2025

Dear Academic Editor,

Thank you and the reviewers for your detailed and thoughtful comments. We have incorporated your feedback into our revised draft. Please find below our responses (in bold) to each point raised by the editor and reviewers.

1. Please ensure that your manuscript meets PLOS ONE's style requirements, including those for file naming. The PLOS ONE style templates can be found at

<https://journals.plos.org/plosone/s/file?id=wjVg/PLOSOne_formatting_sample_main_body.pdf> a nd

https://journals.plos.org/plosone/s/file?id=ba62/PLOSOne_formatting_sample_title_authors_affili ations.pdf. - **We have made the adjustments for file naming and followed the style**

# requirements as per the templates provided.

2. Please include a complete copy of PLOS’ questionnaire on inclusivity in global research in your revised manuscript. Our policy for research in this area aims to improve transparency in the reporting of research performed outside of researchers’ own country or community. The policy applies to researchers who have travelled to a different country to conduct research, research with Indigenous populations or their lands, and research on cultural artefacts. The questionnaire can also be requested at the journal’s discretion for any other submissions, even if these conditions are not met. Please find more information on the policy and a link to download a blank copy of the questionnaire here: [https://journals.plos.org/plosone/s/best-](https://journals.plos.org/plosone/s/best-practices-in-research-reporting) [practices-in-research-reporting](https://journals.plos.org/plosone/s/best-practices-in-research-reporting). Please upload a completed version of your questionnaire as Supporting Information when you resubmit your manuscript.” - **We have uploaded the**

# completed questionnaire and included more information about ethical approval in Kenya.

3. During your revisions, please note that a simple title correction is required: Please change "a randomized controlled trial" to "a cluster randomized controlled trial". Please ensure this is updated in the manuscript file and the online submission information. *-* **We are no longer**

# putting the type of trial into the title of the manuscript. We have changed it to "a cluster randomized controlled trial" in the text.

4. Thank you for stating in your Funding Statement:

“Lalor Foundation and Save the Children (Sweden) supported the program implementation, which was in this study. Both funders had no role of any kind in study design, data collection, analysis, etc., and did not specifically fund the evaluation.”

Please provide an amended statement that declares *all* the funding or sources of support (whether external or internal to your organization) received during this study, as detailed online

in our guide for authors at <http://journals.plos.org/plosone/s/submit-now>. Please also include the statement “There was no additional external funding received for this study.” in your updated Funding Statement.

Please include your amended Funding Statement within your cover letter. We will change the online submission form on your behalf. - **We have now clarified that the study was paid for by the Lalor Foundation, and Save the Children covered the project implementation as part of a larger grant to CSA. We have also added,** **"There was no additional external funding received for this study" into the updated Funding statement and changed the Funding Statement in the cover letter.**

5. We note that the grant information you provided in the ‘Funding Information’ and ‘Financial Disclosure’ sections do not match.

When you resubmit, please ensure that you provide the correct grant numbers for the awards you received for your study in the ‘Funding Information’ section. - **We have now matched the ‘Funding Information’ and ‘Financial Disclosure’ sections. We have a grant number for Lalor Foundation but not for Save the Children. The grant number for Lalor Foundation has been provided.**

6. Thank you for stating the following in the Acknowledgments Section of your manuscript:

“We are grateful for funding provided by the Lalor Foundation and Save the Children (Sweden).“

We note that you have provided additional information within the Acknowledgements Section that is not currently declared in your Funding Statement. Please note that funding information should not appear in the Acknowledgments section or other areas of your manuscript. We will only publish funding information present in the Funding Statement section of the online submission form.

Please remove any funding-related text from the manuscript and let us know how you would like to update your Funding Statement. Currently, your Funding Statement reads as follows:

“Lalor Foundation and Save the Children (Sweden) supported the program implementation, which was in this study. Both funders had no role of any kind in study design, data collection, analysis, etc., and did not specifically fund the evaluation.”

Please include your amended statements within your cover letter; we will change the online submission form on your behalf. - **We have now removed the funding from the**

# Acknowledgements section and anywhere in the manuscript. We have only added

**funding information in the Funding Statement and have added the funding statement in our cover letter.**

7. Please remove all personal information, ensure that the data shared are in accordance with participant consent, and re-upload a fully anonymized data set.

Note: spreadsheet columns with personal information must be removed and not hidden as all hidden columns will appear in the published file.

Additional guidance on preparing raw data for publication can be found in our Data Policy ([https://journals.plos.org/plosone/s/data-availability#loc-human-research-participant-data-and-](https://journals.plos.org/plosone/s/data-availability#loc-human-research-participant-data-and-other-sensitive-data) [other-sensitive-data](https://journals.plos.org/plosone/s/data-availability#loc-human-research-participant-data-and-other-sensitive-data)) and in the following article: [http://www.bmj.com/content/340/bmj.c181.long.](http://www.bmj.com/content/340/bmj.c181.long)

# - We have now ensured that there are no hidden columns and fully anonymized the data set. We have reviewed the additional guidance shared and changed specific health

**service dates to MM/YYYY format.**

8. Please remove your figures from within your manuscript file, leaving only the individual TIFF/EPS image files, uploaded separately. These will be automatically included in the reviewers’ PDF. - **We have now removed the figure from the manuscript and included it only as a separate image file.**
9. Please include captions for your Supporting Information files at the end of your manuscript, and update any in-text citations to match accordingly. Please see our Supporting Information guidelines for more information: <http://journals.plos.org/plosone/s/supporting-information>. - **We have done so for the inclusivity questionnaire.**
10. If the reviewer comments include a recommendation to cite specific previously published works, please review and evaluate these publications to determine whether they are relevant and should be cited. There is no requirement to cite these works unless the editor has indicated otherwise. - **Thank you for this clarification. We did not see any specific citations that we were asked to use. We have provided citations where they flagged as missing.**

Additional Editor Comments (if provided):

Thanks for the important study on an issue that is currently of key need to publish and be most aware for adolescents and youth. There are a few suggestions to improve the manuscript. See what you can do so that it is more readable and consistent with scientific expectations. Please also find a few recommendations in the attached file as well. - **We are glad that the editor**

# considers this an important study that needs to be published. We appreciate that

**recommendations in the attached file and have made the requested changes. We have also sought to make it more readable and rigorous.**

Reviewers' comments:

Reviewer's Responses to Questions

# Comments to the Author

5. Review Comments to the Author

Reviewer #1: The article provides a compelling case for improving SRH access among rural youth, and the AHAP model is clearly impactful. However, the data (2018–2019) predates COVID-19, which likely altered youth behaviour and service access patterns significantly. - **Youth behavior in rural Kenya was indeed altered during the height of the epidemic in**

# 2020-21, when schools were closed and may facilities had skeleton staffs. But starting in late 2021, the situation returned to how it was in 2018-19. Our Kenyan collaborators

**confirm this. We have now added a paragraph about this issue in the paper.**

It would strengthen the article to include a discussion or reflection on how the pandemic may have influenced SRH service delivery and adolescent needs in rural Kenya. - **Please see the comment above. The pandemic has not had lasting effect on youth behavior and needs. There was a rise in unwanted adolescent pregnancies during the Covid period, but that seems to have subsided. We have added a citation to that effect.**

Consider exploring whether AHAP or similar interventions were adapted or sustained during the pandemic, and what lessons were learned. - **AHAP was not sustained during the pandemic. We have now clarified this in the paper.**

The inclusion of more recent data or a follow-up study would greatly enhance the relevance and applicability of your findings in the current context. - **Unfortunately, we don't have data that is more recent. However, rural Kenya changes very slowly over time, so we srongly believe that the data presented in this paper is still relevant.**

Reviewer #2: Line 49-50 provide a citation- **We have now provided a citation to line 49-50.**

In the methods section, the analysis section needs more details to move beyond descriptive statistics - **Our data on use of facilities by youth clients only permits descriptive statistics, but because of the concern of this reviewer we did do generalized linear mixed modelling to test if there was a cluster effect by facilities. We present this new data in the paper**

# and describe the technique.

The analysis is too descriptive given the study design. How did you account for the clustering of observations within groups (clusters), acknowledging that individuals within the same cluster tend to be more similar? Consider more complex models for example Multilevel (Hierarchical) Models (Mixed-Effects Models)- **As mentioned above, we now are presenting generalized**

# linear mixed modelling to take into account cluster effects in the client satisfaction card data.

You could also provide details about future research directions. One approach that could be used is to use Discrete Choice Experiments (DCEs) to elicit participants' preferences and use these to help design interventions to help improve service uptake among the adolescents.-

# Thank you for this suggestion. We have now added a section on future research directions and have included mention of Discrete Choice Experiments.

Thank you again for your consideration of our manuscript. We look forward to proceeding with next steps on this publication.

Sincerely,

Paula Tavrow, PhD

Adjunct Professor, Department of Community Health Sciences UCLA Fielding School of Public Health

[ptavrow@ucla.edu](mailto:ptavrow@ucla.edu)

(310) 435-1444

*(On behalf of: Dr. Paula Tavrow, Krupa Maria Varghese, Albert Obbuyi, and Collins Juma)*
