# Supplementary material for: Increasing young people’s use of sexual and reproductive health services in government health facilities in rural Kenya
Source: PLoS One. 2026 May 4;21(5):e0347329. doi: 10.1371/journal.pone.0347329 (PMC13138647; doi:10.1371/journal.pone.0347329)
Supplement: Attachment — Submitted filename: AHAP review.docx [file pone.0347329.s007.docx]

**An example methods section to report the use of GLME**

A generalized linear mixed effects model was used to assess associations of [independent variables] with [dependent variable], accounting for clustering by [cluster variable, e.g. study sites]. The model contains fixed effects for predictors and a random intercept for clusters in order to capture within-group correlation. This approach enables the modeling of the binary outcome with appropriate consideration of the non-independence of observations within clusters, providing robust estimates of associations while adjusting for potential group-level variability.

**Comments for Table 3 analysis**

When presenting logistic regression results in a table with independent variables in the rows and the dependent variable in the columns, it is important to show the proportions of the dependent variable categories as column percentages. This approach clearly communicates how the outcome varies across different predictor levels and aligns intuitively with the interpretation of odds ratios (ORs), which compare the odds of the outcome across these groups. For independent variables with more than two categories, each category should have a corresponding OR that is compared against a designated reference category. Additionally, an overall p-value from the Wald test can be presented to assess the joint significance of the variable with multiple categories. This overall p-value informs whether the independent variable, taken as a whole, is statistically significantly associated with the outcome. Including these elements in the table—column percentages for the outcome distribution, category-wise ORs relative to a reference, and an overall Wald p-value—ensures a comprehensive, clear, and statistically sound presentation of logistic regression results.

**Reference category:** I recommend that you use the largest-subgroup reference categories to provide stable baselines, reduce variability, and enhance model stability.

**Example table**

| **Variable** | **Total N** | **n(%) Full AHAP** | **Unadjusted**  **Odds Ratio (OR)** | **Adjusted model**  **Odds Ratio (AOR)** | **P-value** |
| --- | --- | --- | --- | --- | --- |
| **Age** |  |  |  |  |  |
| 10-21 | 33859 | 8429(24.9) | 1 | 1 | **0.016** |
| 22-30 | 28801 | 8896(30.9) | 1.24(1.06-1.46) | 1.18(1.03-1.35) |  |
| **Client gender** |  |  |  |  |  |
| Female | 30826 | 4626(15.0) | 1 | 1 |  |
| Male | 18076 | 4626(25.6) | 1.71(1.56-1.86) | 1.63(1.50-1.78) | **<0.001** |
| **Main service desired** |  |  |  |  |  |
| Family planning |  |  | 1.68 (1.24-2.11) |  |  |
| Condoms |  |  | 1 |  | 0.121 |
| Preg test |  |  | 0.98 (0.31-1.21) |  |  |
| Condoms |  |  | 1.05 (0.96-1.87) |  |  |
| VCT |  |  | 0.32 (0.14-0.92) |  |  |
| Counselling only |  |  | 0.57 (0.18-1.10) |  |  |
| Other |  |  | 1.21 (0.95-1.97) |  |  |
