# Supplementary material for: Increasing young people’s use of sexual and reproductive health services in government health facilities in rural Kenya
Source: PLoS One. 2026 May 4;21(5):e0347329. doi: 10.1371/journal.pone.0347329 (PMC13138647; doi:10.1371/journal.pone.0347329)
Supplement: Attachment — Submitted filename: Response to Reviewers Feb 2026.docx [file pone.0347329.s008.docx]

**SECOND REVISION**

**AUTHORS’ RESPONSES**

**We appreciate that reviewer’s careful explanation on how to revise our table showing generalized linear mixed effects, taking into account the effects of facility clusters, in a way that is clearer to the reader. Our Table 4 now uses the format proposed by the reviewer. (We also decided to insert a new Table 3 that compares male and female youth clients, with comprehensive data about the services received and their satisfaction. Then we redid our Table 4 to follow the approach proposed by the reviewer. This way the reader is still able to see the complete data on range of services and various satisfaction items, as well as whether there were differences by type of intervention.)**

**There was only one recommendation the reviewer made that we did not follow in its entirety. The reviewer recommended that we use the largest-subgroup as our reference categories. For some categories it made sense, but not for age (where the largest category was the middle category of 18-21) or for gender (since we wanted to focus our discussion on the female youths—who are often underserved--and felt the male youths should be the reference category). We felt it was intuitively more understandable to readers if the aOR for younger age and female clients was presented as >1 so that they can see the extent to which female youths and young youths (10-17) were more drawn to full AHAP facilities (e.g., twice as likely to visit them) than were males and older clients. (As a test, we did run the GLME using largest-subgroup reference categories and did not find that it made any significant difference in our findings.)**

**____________________________**

**REVIEWER’S COMMENTS**

**An example methods section to report the use of GLME**

A generalized linear mixed effects model was used to assess associations of [independent variables] with [dependent variable], accounting for clustering by [cluster variable, e.g. study sites]. The model contains fixed effects for predictors and a random intercept for clusters in order to capture within-group correlation. This approach enables the modeling of the binary outcome with appropriate consideration of the non-independence of observations within clusters, providing robust estimates of associations while adjusting for potential group-level variability.

**Comments for Table 3 analysis**

When presenting logistic regression results in a table with independent variables in the rows and the dependent variable in the columns, it is important to show the proportions of the dependent variable categories as column percentages. This approach clearly communicates how the outcome varies across different predictor levels and aligns intuitively with the interpretation of odds ratios (ORs), which compare the odds of the outcome across these groups. For independent variables with more than two categories, each category should have a corresponding OR that is compared against a designated reference category. Additionally, an overall p-value from the Wald test can be presented to assess the joint significance of the variable with multiple categories. This overall p-value informs whether the independent variable, taken as a whole, is statistically significantly associated with the outcome. Including these elements in the table—column percentages for the outcome distribution, category-wise ORs relative to a reference, and an overall Wald p-value—ensures a comprehensive, clear, and statistically sound presentation of logistic regression results.

**Reference category:** I recommend that you use the largest-subgroup reference categories to provide stable baselines, reduce variability, and enhance model stability.

**Example table**

| **Variable** | **Total N** | **n(%) Full AHAP** | **Unadjusted**  **Odds Ratio (OR)** | **Adjusted model**  **Odds Ratio (AOR)** | **P-value** |
| --- | --- | --- | --- | --- | --- |
| **Age** |  |  |  |  |  |
| 10-21 | 33859 | 8429(24.9) | 1 | 1 | **0.016** |
| 22-30 | 28801 | 8896(30.9) | 1.24(1.06-1.46) | 1.18(1.03-1.35) |  |
| **Client gender** |  |  |  |  |  |
| Female | 30826 | 4626(15.0) | 1 | 1 |  |
| Male | 18076 | 4626(25.6) | 1.71(1.56-1.86) | 1.63(1.50-1.78) | **<0.001** |
| **Main service desired** |  |  |  |  |  |
| Family planning |  |  | 1.68 (1.24-2.11) |  |  |
| Condoms |  |  | 1 |  | 0.121 |
| Preg test |  |  | 0.98 (0.31-1.21) |  |  |
| Condoms |  |  | 1.05 (0.96-1.87) |  |  |
| VCT |  |  | 0.32 (0.14-0.92) |  |  |
| Counselling only |  |  | 0.57 (0.18-1.10) |  |  |
| Other |  |  | 1.21 (0.95-1.97) |  |  |
